# A coupled mitral valve—left ventricle model with fluid–structure interaction

**DOI:** 10.1016/j.medengphy.2017.06.042

**Published:** 2017-07-25

**Authors:** Hao Gao, Liuyang Feng, Nan Qi, Colin Berry, Boyce E. Griffith, Xiaoyu Luo

**Affiliations:** a School of Mathematics and Statistics, University of Glasgow, Glasgow, UK; b Institute of Cardiovascular and Medical Science, University of Glasgow, Glasgow, UK; c Departments of Mathematics and Biomedical Engineering and McAllister Heart institute, University of North Carolina, Chapel Hill, NC, USA

**Keywords:** Mitral valve, Left ventricle, Fluid–structure interaction, Immersed boundary method, Finite element method, Soft tissue mechanics

## Abstract

Understanding the interaction between the valves and walls of the heart is important in assessing and subsequently treating heart dysfunction. This study presents an integrated model of the mitral valve (MV) coupled to the left ventricle (LV), with the geometry derived from in vivo clinical magnetic resonance images. Numerical simulations using this coupled MV-LV model are developed using an immersed boundary/finite element method. The model incorporates detailed valvular features, left ventricular contraction, nonlinear soft tissue mechanics, and fluid-mediated interactions between the MV and LV wall. We use the model to simulate cardiac function from diastole to systole. Numerically predicted LV pump function agrees well with in vivo data of the imaged healthy volunteer, including the peak aortic flow rate, the systolic ejection duration, and the LV ejection fraction. In vivo MV dynamics are qualitatively captured. We further demonstrate that the diastolic filling pressure increases significantly with impaired myocardial active relaxation to maintain a normal cardiac output. This is consistent with clinical observations. The coupled model has the potential to advance our fundamental knowledge of mechanisms underlying MV-LV interaction, and help in risk stratification and optimisation of therapies for heart diseases.

## 1. Introduction

The mitral valve (MV) has a complex structure that includes two distinct asymmetric leaflets, a mitral annulus, and chordae tendineae that connect the leaflets to papillary muscles that attach to the wall of the left ventricle (LV). MV dysfunction remains a major medical problem because of its close link to cardiac dysfunction leading to morbidity and premature mortality [1].

Computational modelling for understanding MV mechanics promises more effective MV repairs and replacement [2–5]. Biomechanical MV models have been developed for several decades, starting from the simplified two-dimensional approximation to three-dimensional models, and to multi-physics/-scale models [6–12]. Most previous studies were based on structural and quasi-static analysis applicable to a closed valve [13]; however, MV function during the cardiac cycle cannot be fully assessed without modelling the ventricular dynamics and the fluid-structure interaction (FSI) between the MV, ventricles, and the blood flow [13,14].

Because of the complex interactions between the MV, the sub-mitral apparatus, the heart walls, and the associated blood flow, very few modelling studies have been carried out that integrate the MV and ventricles in a single model [15–17]. Kunzelman, Einstein, and co-workers first simulated normal and pathological mitral function [18–20] with FSI using LS-DYNA (Livermore Software Technology Corporation, Livermore, CA, USA) by putting a MV into a straight tube. Using a similar modelling approach, Lau et al. [21] compared MV dynamics with and without FSI, and they found that valvular closure configuration is different when using the FSI MV model. Similar findings were reported by Toma et al. [22]. Over the last few years, there have also been a number of FSI valvular models using immersed boundary (IB) method to study the flow across the MV [23–25]. In a series of studies, Toma et al. [22,26,27] developed an FSI MV model based on in vitro MV experimental system to study the function of the chordal structure, and good agreement was found between the computational model and in vitro experimental measurements. However, none of the aforementioned MV models accounted for the MV interaction with the LV dynamics. Indeed, Lau et al. [21] found that even with a fixed U-shaped ventricle, the flow pattern was substantially different from that estimated using a tubular geometry. Despite the advancements in computational modelling of individual MV [12,13] and LV models [28–30], it remains challenging to develop an integrated MV–LV model that includes the strong coupling between the valvular deformation and the blood flow. Reasons for this include limited data for model construction, difficult choices of boundary conditions, and large computational resources required by these simulations.

Wenk et al. [15] reported a purely structural MV-LV model using LS-DYNA that included the LV, MV, and chordae tendineae. This model was later extended to study MV stress distributions using a saddle-shaped and asymmetric mitral annuloplasty ring [16]. A more complete whole-heart model was recently developed using a human cardiac function simulator in the Dassault Systèmes *Living Heart* project [17], which included four ventricular chambers, cardiac valves, electrophysiology, and detailed myofibre and collagen architecture. Using the same simulator, effects of different mitral annulus ring were studied by Rausch et al. [31], However, this simulator does not account for detailed FSI.

The earliest valve-heart coupling model that includes FSI is credited to Peskin and McQueen’s pioneering work in the 1970s [32–34] using the classical IB approach [35]. Using this same method, Yin et al. [36] investigated fluid vortices associated with the LV motion as a prescribed moving boundary. Recently, Chan-dran and Kim [37] reported a prototype FSI MV dynamics in a simplified LV chamber model during diastolic filling using an immersed interface-like approach. One of the key limitations of these coupled models is the simplified representation of the biomechanics of the LV wall. To date, there has been no work reported a coupled MV-LV model which has full FSI and based on realistic geometry and experimentally-based models of soft tissue mechanics.

This study reports an integrated MV-LV FSI model derived from in vivo images of a healthy volunteer. Although some simplifications are made, this is the first three-dimensional FSI MV-LV model that includes MV dynamics, LV contraction, and experimentally constrained descriptions of nonlinear soft tissue mechanics. This work is built on our previous models of the MV [24,25] and LV [29,38]. The model is implemented using a hybrid immersed boundary method with finite element elasticity (IB/FE) [39].

## 2. Methodology

### 2.1. MV-LV model construction

**Fig. 1.**
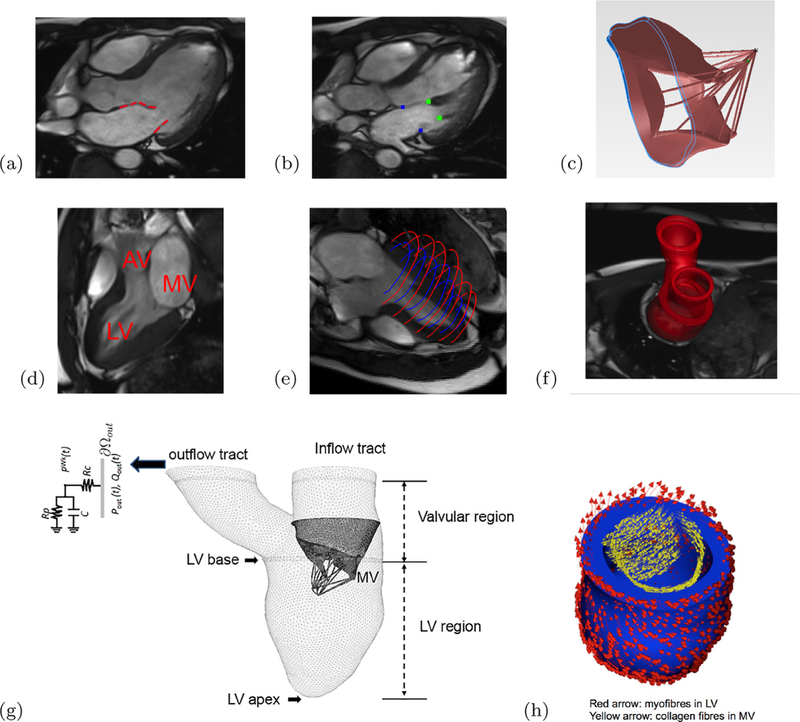
The CMR-derived MV-LV model, (a) The MV leaflets were segmented from a stack of MR images of a volunteer at early-diastole, (b) positions of the papillary muscle heads and the annulus ring, (c) reconstructed MV geometry with chordae, (d) an MR image showing the LV and location of the outflow tract (AV) and inflow tract (MV), (e) the LV wall delineation from short and long axis MR images, (f) the reconstructed LV model, in which the LV model is divided into four part: the LV region bellow the LV base, the valvular region, and the inflow and outflow tracts, (g) the coupled MV LV model, and (h) the rule-based fibre orientations in the LV and the MV. (For interpretation of the references to colour in the text, the reader is referred to the web version of this article.)

A cardiac magnetic resonance (CMR) study was performed on a healthy volunteer (male, age 28). The study was approved by the local NHS Research Ethics Committee, and written informed consent was obtained before the CMR scan. Twelve imaging planes along the LV outflow tract (LVOT) view were imaged to cover the whole MV region shown in Fig. 1(a). LV geometry and function were imaged with conventional short-axis and long-axis cine images. The parameters for the LVOT MV cine images were: slice thickness: 3 mm with 0 gap; in-plane pixel size: 0.7 × 0.7 mm^2^; field of view: 302 × 400mm^2^; frame rate: 25 per cardiac cycle. Short-axis cine images covered the LV region from the basal plane to the apex, with slice thickness: 7 mm with 3 mm gap; in-plane pixel size: 1.3 × 1.3 mm^2^; frame rate: 25 per cardiac cycle.

The MV geometry was reconstructed from LVOT MV cine images at early-diastole, just after the MV opens. The leaflet boundaries were manually delineated from MR images, as shown in Fig. 1(a), in which the heads of papillary muscle and the annulus ring were identified as shown in Fig. 1(b). The MV geometry and its sub-valvular apparatus were reconstructed using SolidWorks (Dassault Systèmes SolidWorks Corporation, Waltham, MA, USA). Because it is difficult to see the chordal structural in the CMR, we modelled the chordae structure using sixteen evenly distributed chordae tendineae running through the leaflet free edges to the annulus ring, as shown in Fig. 1(c), following prior studies [24,25]. In a similar approach to the MV reconstruction, the LV geometry was reconstructed from the same volunteer at early-diastole by using both the short-axis and long-axis cine images [29,40]. Fig. 1(d) shows the inflow and outflow tracts in one MR image. The LV wall was assembled from the short-/long-axis MR images (Fig. 1(e)) to form the three dimensional reconstruction (Fig. 1(f)). The LV model was divided into four regions: the LV wall, the valvular region, and the inflow and the outflow tracts, as shown in Fig. 1(g).

The MV model was mounted into the inflow tract of the LV model according to the relative positions derived from the MR images in Fig. 1(g). The left atrium was not reconstructed but modelled as a tubular structure, and the gap between the MV annulus ring and the LV model was filled using a housing disc structure. A three-element Windkessel model was attached to the outflow tract of the LV model to provide physiological pressure boundary conditions when the LV is in systolic ejection [40]. The chordae were not directly attached to the LV wall since the papillary muscles were not modelled, as in our previous study [25]. The myocardium has a highly layered myofibre architecture, which is usually described using a fibre-sheet-normal (**f**, **s**, **n**) system. A rule-based method was used to construct the myofibre orientation within the LV wall. The myofibre angle was assumed to rotate from −60° to 60° from endocardium to epicardium, represented by the red arrows in Fig. 1(h). In a similar way, the collagen fibres in the MV leaflets were assumed to be circumferentially distributed, parallel along the annulus ring, represented by the yellow arrows in Fig. 1(h).

### 2.2. IB/FE framework

The coupled MV-LV model employs an Eulerian description for the blood, which is modelled as a viscous incompressible fluid, along with a Lagrangian description for the structure. The fixed physical coordinates are **x** = (x_1_,x_2_,x_3_) ∈ Ω, and the Lagrangian reference coordinate system is **X** = (X_1_,X_2_,X_3_) ∈ *U*. The exterior unit normal along *∂U* is ***N***(***X***). Let ***χ***(***X***, *t*) denote the physical position of any material point ***X*** at time t, so that ***χ*** (*U*, *t*) = Ω^s^ (*t*) is the physical region occupied by the immersed structure. The IB/FE formulation of the FSI system reads

(1) $$\rho \left(\frac{\partial u}{\partial t}(x,t)+u(x,t)\cdot \nabla u(x,t)\right)=-\nabla p(x,t)+\mu \nabla^{2}u(x,t)+f^{\text{s}}(x,t),$$

(2) ∇⋅u(x,t)=0,

(3) $$f^{\text{s}}(x,t)=\int_{U}\nabla \cdot {\mathbb{P}}^{\text{s}}(X,t)\;\delta (x-\chi (X,t))\text{ d}X-\int_{\partial U}{\mathbb{P}}^{\text{s}}(X,t)N(X)\;\delta (x-\chi (X,t))\text{ d}A(X),$$

(4) $$\frac{\partial \chi }{\partial t}(X,t)=\int_{\Omega }u(x,t)\delta (x-\chi (X,t))\text{ d}x,$$

where *ρ* is the fluid density, *μ* is the fluid viscosity, ***u*** is the Eulerian velocity, *p* is the Eulerian pressure, and ***f***^s^ is the Eulerian elastic force density, which is determined from the deformed immersed structure and its active contraction. Note the IB formulation employs a common momentum equation for both the fluid and the solid, in which the additional solid stresses are accounted for by ***f***^s^ in Eq. (1). Interactions between the Lagrangian and Eulerian fields are achieved by integral transforms with a Dirac delta function kernel δ(**x**)[35], in Eqs. (3) and (4). Different from the classical IB approach [35], here the elastic force density ***f***^s^ is determined from the first Piola-Kirchoff stress tensor ${\mathbb{P}}^{\text{s}}$. This allows the solid deformations to be described using a nonlinear hyperelastic constitutive law; see Section 2.3 below. For more details of the hybrid IB/FE framework, readers are referred to the paper by Griffith and Luo [39].

### 2.3. Soft tissue mechanics

The total Cauchy stress (**σ**) in the coupled MV-LV system is

(5) $$\sigma (x,t)=\{\begin{array}{ll} \sigma^{\text{f}}(x,t)+\sigma^{\text{s}}(x,t) & \text{ for }x\in \Omega^{\text{s}},\\ \sigma^{\text{f}}(x,t) & \text{ otherwise, } \end{array}$$

where **σ**^f^ is the fluid-like stress tensor, defined as

(6) $$\sigma^{\text{f}}(x,t)=-pI+\mu \left[\nabla u+{\left(\nabla u\right)}^{T}\right].$$

**σ**^s^ is the solid stress tensor obtained from the nonlinear soft tissue constitutive laws. The first Piola-Kirchhoff stress tensor ${\mathbb{P}}^{\text{s}}$ in Eq. (3) is related to **σ**^s^ through

(7) $${\mathbb{P}}^{\text{s}}=J\sigma^{\text{s}}F^{-T},$$

in which F=∂χ/∂X is the deformation gradient and J=det(F).

In the MV-LV model, we assume the structure below the LV base is contractile (Fig. 1(g)), the regions above the LV basal plane, including the MV and its apparatuses, are passive. Namely,

(8) $${\mathbb{P}}^{\text{s}}=\{\begin{array}{ll} {\mathbb{P}}^{\text{p}}+{\mathbb{P}}^{\text{a}} & \text{ below the basal plane,}\\ {\mathbb{P}}^{{}^{\text{p}}} & \text{ above the basal plane,} \end{array}$$

where ${\mathbb{P}}^{\text{a}}$ and ${\mathbb{P}}^{\text{p}}$ are the active and passive Piola-Kirchhoff stress tensors, respectively. The MV leaflets are modelled as an incompressible fibre-reinforced material with the strain energy function

(9) $$W_{\text{MV}}=C_{1}\left(I_{1}-3\right)+\frac{a_{\text{v}}}{2b_{\text{v}}}\left(\text{exp}\left[b_{\text{v}}{\left(\text{max}\left(I_{\text{f}}^{\text{c}},1\right)-1\right)}^{2}\right]-1\right),$$

in which $I_{1}=\text{trace}(\mathbb{C})$ is the first invariant of the right Cauchy-Green deformation tensor $\mathbb{C}=F^{T}F$, $I_{\text{f}}^{\text{c}}=f_{0}^{\text{c}}\cdot \left(\mathbb{C}f_{0}^{\text{c}}\right)$ is the squared stretch along the collagen fibre direction, and $f_{0}^{\text{c}}$ denotes the collagen fibre orientation in the reference configuration. The max (•) function ensures the embedded collagen network only bears loads when stretched, but not in compression. *C*_1_, *a*_v_, and *b*_v_ are material parameters adopted from a prior study [25] and listed in Table 1. The passive stress tensor ${\mathbb{P}}^{\text{p}}$ in the MV leaflets is then

(10) $${\mathbb{P}}^{\text{p}}=\frac{\partial W_{\text{MV}}}{\partial F}-C_{1}F^{-T}+\beta_{\text{s}}\text{ log}\left(I_{3}\right)F^{-T},$$

where $I_{3}=\text{det}(\mathbb{C})$, and *β*_s_ is the bulk modulus for ensuring the incompressibility of immersed solid, and the pressure-like term $C_{1}F^{-T}$ ensures the elastic stress response is zero when F=I.

We model the chordae tendineae as a neo-Hookean material,

(11) $$W_{\text{chordae }}=C\left(I_{1}-3\right),$$

where *C* is the shear modulus. We further assume *C* is much larger in systole when the MV is closed than in diastole when the valve is opened to account for the effects of papillary muscle contraction. Values of C are listed in Table 1. ${\mathbb{P}}^{\text{p}}$ for the chordae tendineae is similarly derived as in Eq. (10).

The passive response of the LV myocardium is described using the Holzapfel-Ogden model [41],

(12) $$W_{\text{myo}}=\frac{a}{2b}\text{exp}\left[b\left(I_{1}-3\right)\right]+\sum_{i=\text{f},\text{s}}\frac{a_{i}}{2b_{i}}\left\{\text{exp}\left[b_{i}{\left(\text{max}\left(I_{4i},1\right)-1\right)}^{2}\right]-1\right\}+\frac{a_{\text{fs}}}{2b_{\text{fs}}}\left\{\text{exp}\left[b_{\text{fs}}{\left(I_{\text{8fs}}\right)}^{2}\right]-1\right\},$$

in which *a, b, a*_f_, *b*_f_*, a*_s_*, b*_*s*_*, a*_fS_, *b*_fs_ are the material parameters, *I*_4f,_ *I*_4s_and *I*_8fs_ are the strain invariants related to the myofibre orientations. Denoting the myofibre direction in the reference state *f*_0_ and the sheet direction by *s*_0_ we have

(13) $$I_{4\text{f}}=f_{0}\cdot \left(\mathbb{C}f_{0}\right),I_{4\text{s}}=s_{0}\cdot \left(\mathbb{C}s_{0}\right),\text{ and }I_{8\text{f}s}=f_{0}\cdot \left(\mathbb{C}s_{0}\right).$$

The myocardial active stress is defined as

(14) $${\mathbb{P}}^{\text{a}}=J\;T\;F\;f_{0}⊗f_{0},$$

where *T* is the active tension described by the myofilament model of Niederer et al. [42], using a set of ordinary differential equations involving the intracellular calcium transient (Ca^2+^), sarcomere length, and the active tension at the resting sarcomere length (*T*^req^).

The constitutive parameters in Eqs. (9), (11) and (12), summarised in Table 1, are obtained from our previous studies [25,29] which showed that the simulated MV and LV dynamics matched the in vivo measurements well. In the active contraction model (Eq. (14)), we adopted the same parameters as used by Niederer et al. [42], except that *T*^req^ is set to 225 kPa to match the contractions observed in the imaged volunteer.

### 2.4. Boundary conditions and model implementation

Because only the myocardium below the LV basal plane contracts, we fix the LV basal plane along the circumferential and longitudinal displacements, but allow the radial expansion. The myocardium below the LV basal plane is left free to move. The valvular region is assumed to be much softer than the LV region. In diastole, a maximum displacement of 6 mm is allowed in the valvular region using a tethering force. In systole, the valve region is gradually pulled back to the original position. The inflow and outflow tracts are fixed. Because the MV annulus ring is attached to a housing structure which is fixed, no additional boundary conditions are applied to the MV annulus ring. Fluid boundary conditions are applied to the top planes of the inflow and outflow tracts. The function of the aortic valve is simply modelled as: the aortic valve is either fully opened or fully closed, determined by the pressure difference between the values inside the LV chamber and the aorta. After end-diastole, the LV region will contract simultaneously triggered by a spatially homogeneously prescribed intracellular Ca^2+^ transient [29], as shown in Fig.3. The flow boundary conditions in a cardiac cycle are summarised below.

- **Diastolic filling:** A linearly ramped pressure from 0 to a population-based end-diastolic pressure (EDP=8 mmHg) is applied to the inflow tract over 0.8 s, which is slightly longer than the actual diastolic duration of the imaged volunteer (0.6 s). In diastole about 80% of diastolic filling volume is due to the “sucking” effect of the left ventricle in early-diastole [43]. This negative pressure field inside the LV cavity is due to the myocardial relaxation. We model this “sucking” effect using an additional pressure loading applied to the endocardial surface, denoted as *P*_endo_, which is linearly ramped from 0 to 12 mmHg over 0.4 s, and then linearly decreased to zero at end-diastole. The value of *P*_endo_ is chosen by matching the simulated end-diastolic volume to the measured data from CMR images. Blood flow is not allowed to move out of the LV cavity through the inflow tract in diastole. Zero flow boundary conditions are applied to the top plane of the outflow tract.
- **Iso-volumetric contraction:** Along the top plane of the inflow tract, the EDP loading is maintained, and the flow is controlled by the MV leaflet dynamics. Note during the iso-volumetric contraction, regurgitation may occur due to the MV closure action and the dysfunction of the MV apparatus. However, a small backflow before the MV is fully closed may be deemed normal [44]. Zero flow boundary conditions are retained for the outflow tract. The duration of the iso-volumetric contraction is determined by the myocardial contraction and ends when the aortic valve opens. The aortic valve opens when the LV pressure is higher than the pressure in the aorta, which is initially set to be the cuff-measured diastolic pressure in the brachial artery, 85 mmHg.
- **Systolic ejection:** When the aortic valve opens, a three-element Windkessl model is coupled to the top plane of the outflow tract to provide afterload. The volumetric flow rates across the top plane of the outflow tract is calculated from the three-dimensional MV-LV model, and fed into the Windkessel model [45], which returns an updated pressure for the outflow tract in the next time step. The systolic ejection phase ends when the left ventricle cannot pump any flow through the outflow tract, and the Windkessel model is detached.
- **Iso-volumetric relaxation:** Zero flow boundary conditions are applied to both the top planes of the outflow and inflow tracts until the total cycle ends at 1.2 s.

The coupled MV-LV model is immersed in a 17 cm × 16 cm × 16 cm fluid box. A basic time step size Δ*t*_0_ = 1.22 × 10^−4^ s is used in the diastolic and relaxation phases, a reduced time step size (0.25 Δ*t*_0_) is used in the early systole with a duration of 0.1 s, and an even smaller time step of 0.125 Δ*t*_0_ is used in the remainder of the systolic phase. Because explicit time stepping is used in the numerical simulations [39], we need to use a time step size small enough to avoid numerical instabilities, particularly during the systolic phase to resolve the highly dynamic LV deformation.

The MV-LV model is implemented using the open-source IBAMR software framework (https://github.com/IBAMR/IBAMR), which provides an adaptive and distributed-memory parallel implementation of the IB methods. IBAMR leverages functionality provided by other freely available software libraries, including SAMRAI (https://computation.llnl.gov/casc/SAMRAI), libMesh (https://libmesh.github.io), PETSc (http://www.mcs.anl.gov/petsc), and *hypre* (http://www.llnl.gov/CASC/hypre). All simulations were carried out on a Linux workstation at the University of Glasgow with 10 Intel(R) Xeon(R) CPU cores (2.65 GHz) and 32 GB RAM. The simulation time for one cardiac cycle is around 240 h (10 days).

## 3. Results

### 3.1. Pump function

**Fig. 2.**
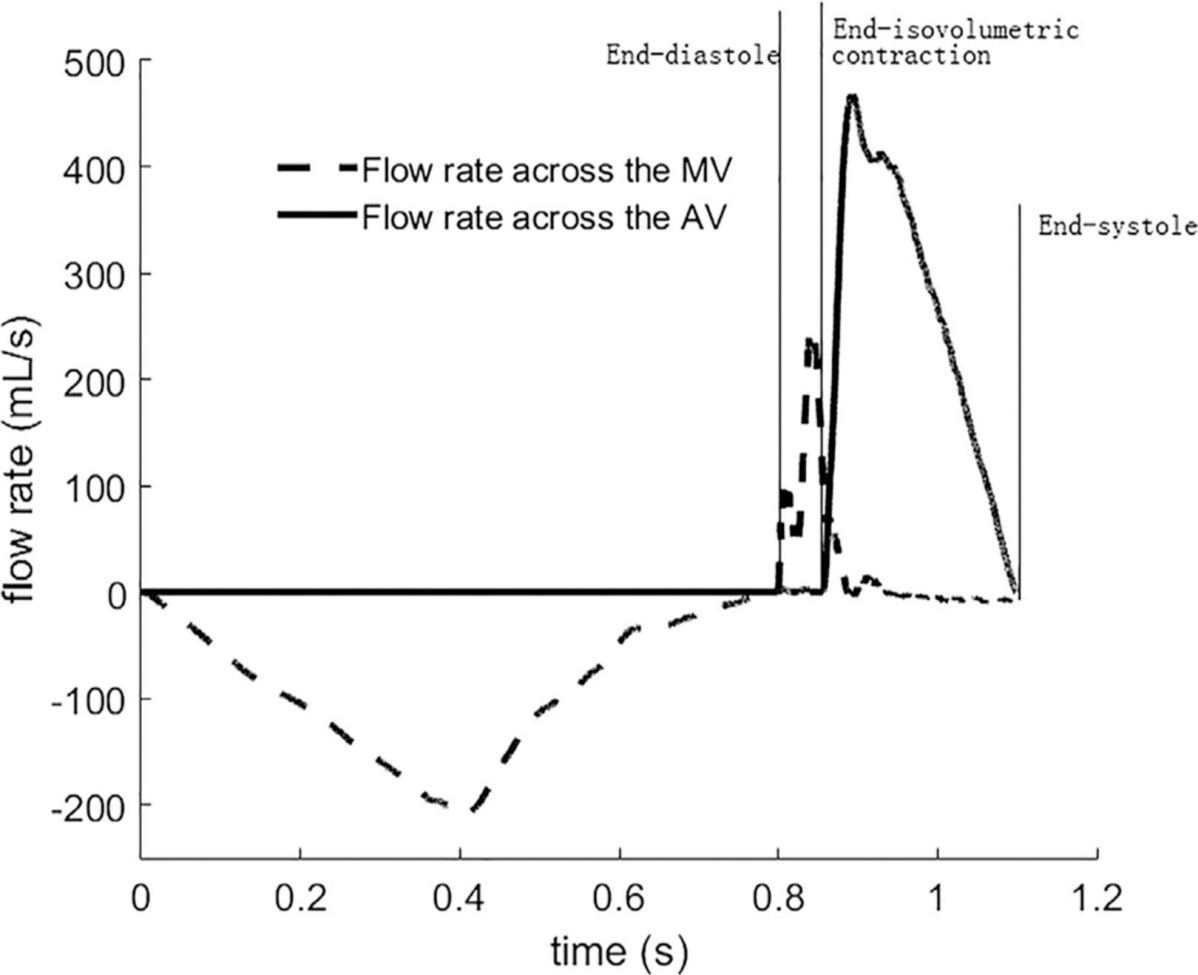
Flow rates across the AV and MV from diastole to systole. Diastolic phase 0 s to 0.8 s; systolic phase: 0.8 s and onwards. Positive flow rate indicates the blood flows out of the LV chamber.

Fig. 2 shows the computed volumetric flow rates through the MV and the AV from beginning of diastole to end-systole. In diastole, the volumetric flow rate through the MV linearly increases with increased pressure loading in the endocardial surface, with a maximum value of 210mL/s at 0.4 s. Diastolic filling is maintained by the increased pressure in the inflow tract, but with decreased flow rates until end of diastole at 0.8 s. The negative flow rate in Fig. 2 indicates the flow is entering the LV chamber. After end-diastole, the myocardium starts to contract, and the central LV pressure increases until it exceeds the aortic pressure (initially set to be 85 mmHg) at 0.857 s. During iso-volumetric contraction, the MV closes with a total closure regurgitation flow of 7.2 mL, around 10% of the total filling volume, which is comparable to the value reported by Laniado et al. [44]. There is only minor regurgitation across the MV during systolic ejection after the iso-volumetric contraction phase. Blood is then ejected out of the ventricle through the AV, and the flow rate through the AV during systole reaches a peak value of 468 mL/s (Fig. 2) (the CMR measured value is 498 mL/s). The total ejection duration is 243 ms (the measured duration is 300 ms) with a stroke volume of 63.2 mL. The total blood ejected out of the LV chamber, including the regurgitation through the MV, is 72.1 mL. This corresponds to an ejection fraction of 51%, and is close to the CMR measured value of 57%.

**Fig. 3.**
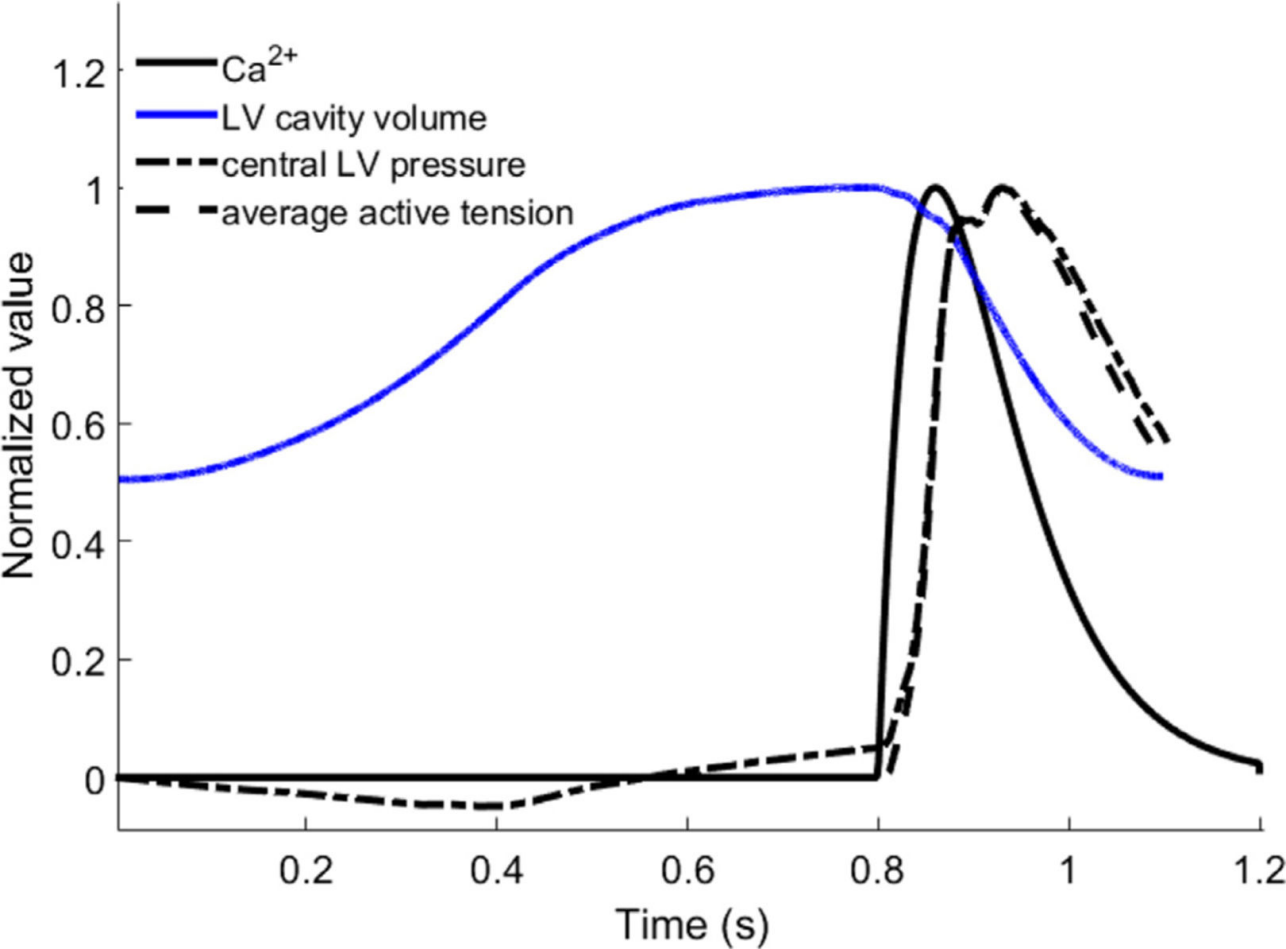
Normalised intracellular Ca^2+^, LV cavity volume, central LV pressure, and average myocardial active tension. All curves are normalised to their own maximum values, which are: 1 μMol for Ca^2+^, 145 mL for LV cavity volume, 162 mmHg for central LV pressure, and 96.3 kPa for average myocardial active tension.

Fig. 3 shows the profiles of the normalised intracellular Ca^2+^, LV cavity volume, central LV pressure, and the average myocardial active tension from diastole to systole. Until mid-diastole (0 s to 0.56 s), the central LV pressure is negative, and the associated diastolic filling volume is around 65 mL, which is 90% of the total diastolic filling volume. In late-diastole, the LV pressure becomes positive. There is a delay between the myocardial active tension and the intracellular Ca^2^+ profile, but the central LV pressure follows the active tension closely throughout the cycle as shown in

Fig. 3. The peak systolic LV pressure is 162 mmHg, comparable to the cuff-measured peak blood pressure 150 mmHg.

### 3.2. Intracardiac flow pattern

**Fig. 4.**
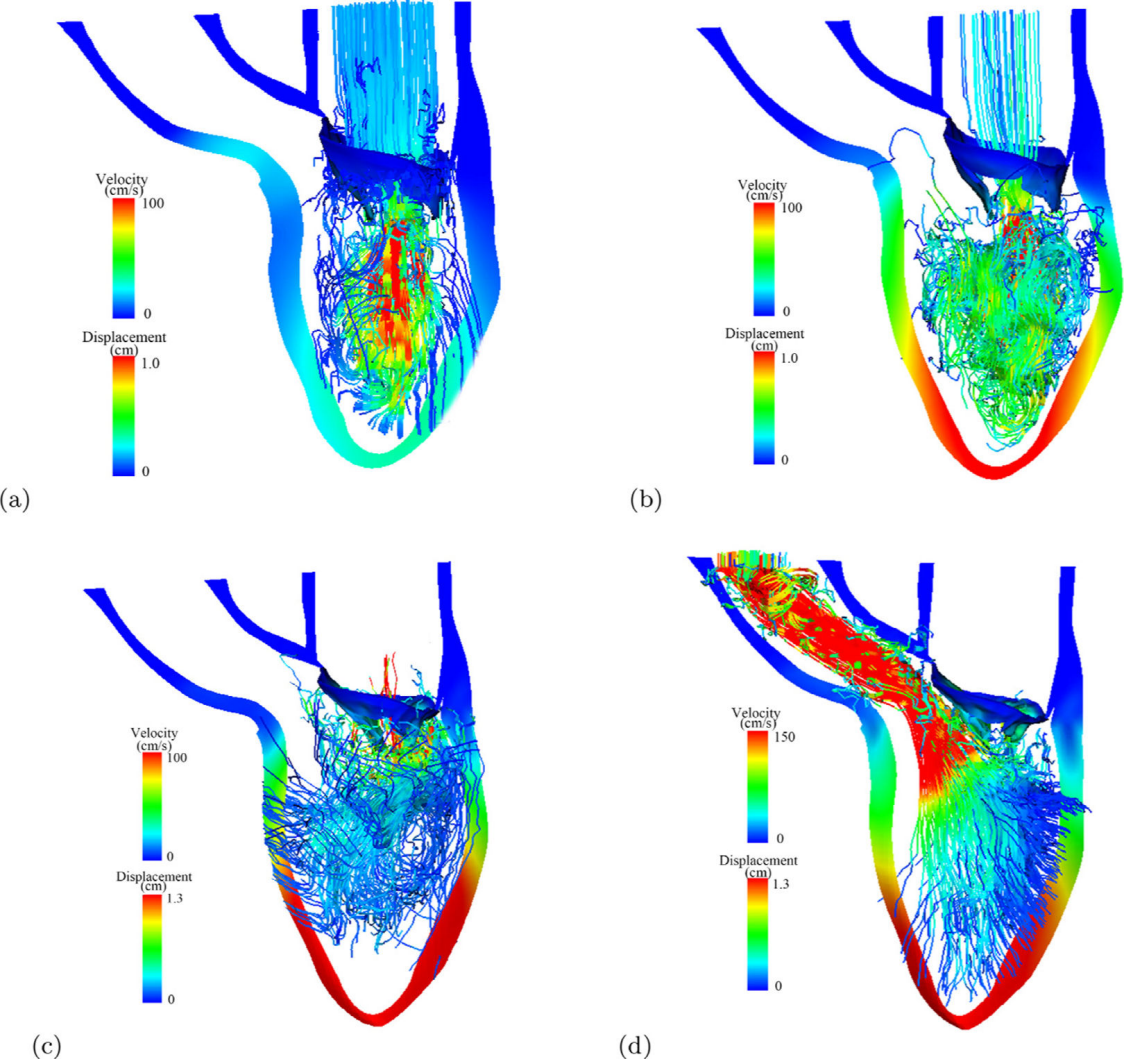
Streamlines in the MV-LV model at early-diastolic filling (a), late-diastolic filling (b), when isovolumetric contraction ends (c), and at the mid-systole. Streamline are coloured by velocity magnitude, the LV wall and MV are coloured by the displacement magnitude. Red: high; blue: low. (For interpretation of the references to colour in this figure legend, the reader is referred to the web version of this article.)

Fig. 4(a–d) shows the streamlines at early-diastolic filling, late-diastolic filling, when the MV is closing (iso-volumetric contraction), and mid-systolic ejection when the left ventricle is ejecting, During the diastolic filling (Fig. 4(a)), the blood flows directly through the MV into the LV chamber towards the LV apex, in late-diastole in Fig. 4(b), the flow pattern becomes highly complex. When iso-volumetric contraction ends, the MV is pushed back towards the left atrium. In mid-systole, the blood is pumped out of the LV chamber through the aortic valve into the systemic circulation, forming a strong jet as shown in Fig. 4(d).

### 3.3. MV and myocardial dynamics

**Fig. 5.**
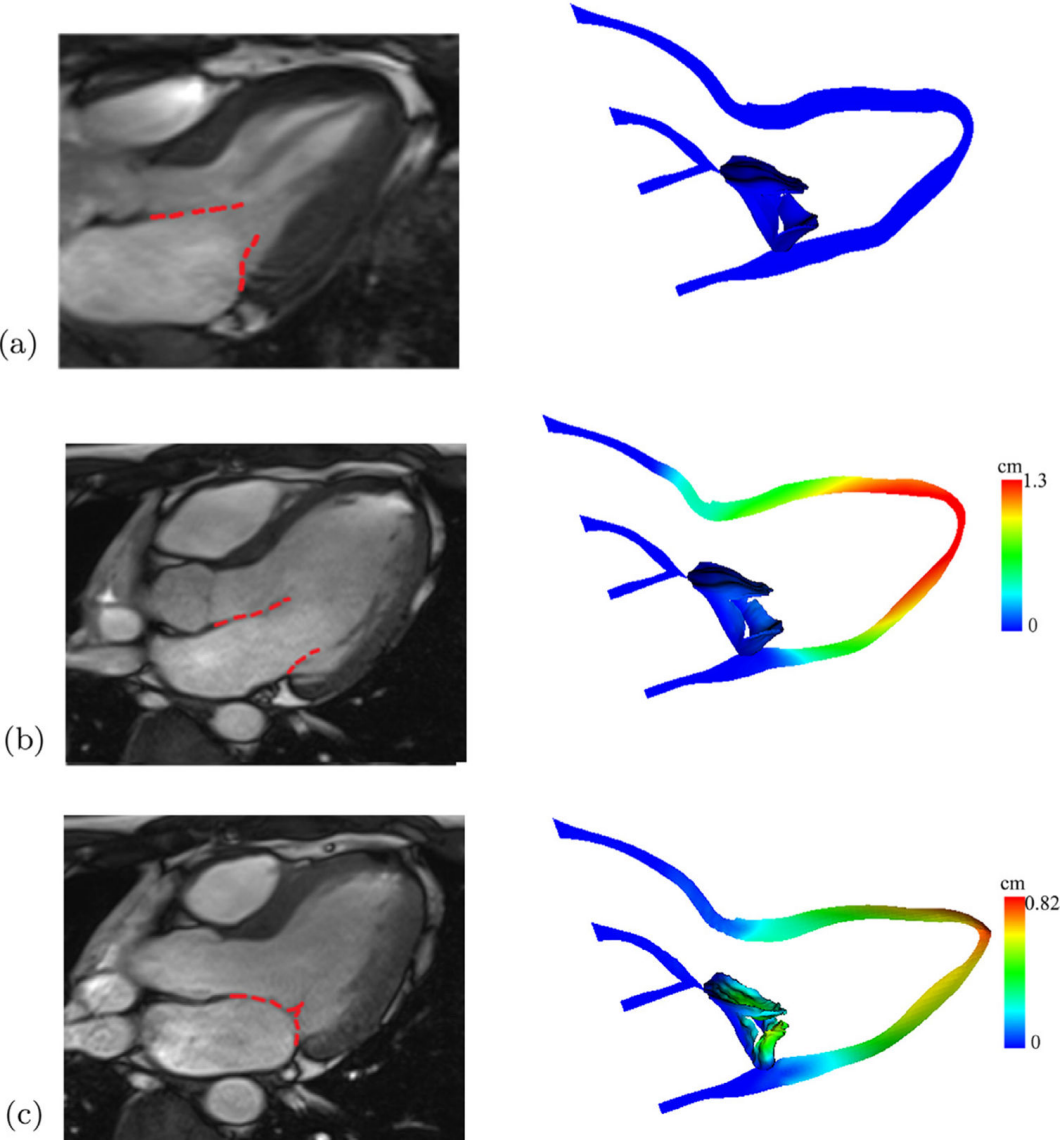
Comparisons between the MV and LV structures at (a) reference configuration, (b) end-diastole, and (c) end-systole, and the corresponding CMR cine images (left). Coloured by the displacement magnitude. (For interpretation of the references to colour in this figure legend, the reader is referred to the web version of this article.)

Fig. 5 shows the deformed MV leaflets along with the corresponding CMR cine images at early-diastole (the reference state), end-diastole, and mid-systole. In general, the in vivo MV and LV dynamics from diastole to systole are qualitatively captured well by the coupled MV-LV model. However, a discrepancy is observed during the diastolic filling, when the MV orifice in the model is not opened as widely as in the CMR cine image (Fig. 5(b)). In addition, the modelled MV leaflets have small gaps near the commissure areas even in the fully closure state. This is partially caused by the finite size of the regularised delta function at the interface and uncertainties in MV geometry reconstruction using CMR images.

**Fig. 6.**
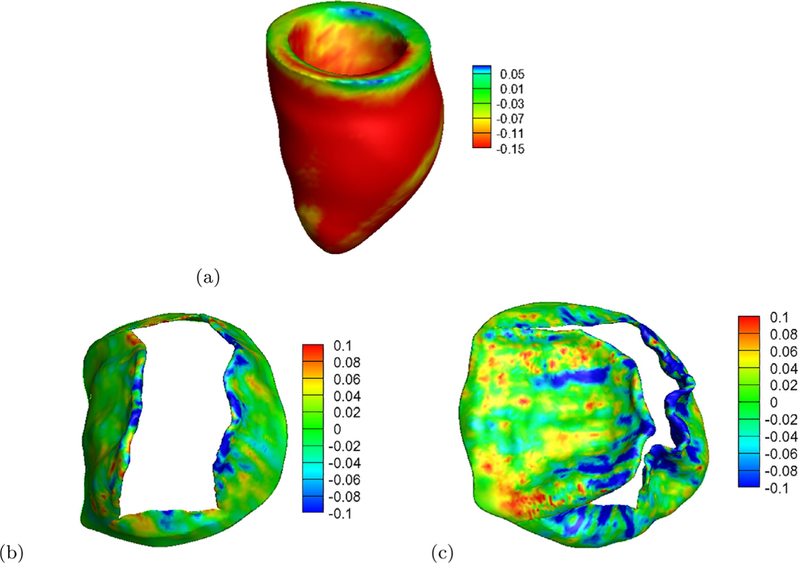
Distributions of fibre strain in the left ventricle at end-systole (a), in the MV at end-diastole (b) and end-systole (c). (For interpretation of the references to colour in this figure legend, the reader is referred to the web version of this article.)

The LV systolic strain related to end-diastole is shown in Fig. 6(a), which is negative throughout most of the region except near the basal plane, where the LV motion is artificially constrained in the model. The average myocardial strain along myofibre direction is −0.162± 0.05, which lies in the normal range of healthy subjects [46]. Fig. 6(b) is the fibre strain in the MV leaflets at end-diastole, the leaflets are mostly slightly stretched during the diastolic filling. In systole, because of the much higher pressure in the LV, the leaflets are pushed towards the left atrium side as shown in Fig. 6(c). Near the leaflet tip and the commissiour areas, the leaflets are highly compressed, while in the trigons near the annulus ring, the leaflet is stretched.

### 3.4. Effects of P_endo_ on pump function

From Fig. 3, it is clear that that the applied endocardial pressure (P_endo_) creates a negative pressure inside the LV chamber, similar to the effects of the myocardial active relaxation. We further investigate how *P*_endo_ affects the MV-LV dynamics by varying its value from 8 mmHg to 16 mmHg, and the effects without *P*_endo_ but with an increased EDP from 8 mmHg to 20 mmHg. We observe that with an increased *P*_endo_, the peak flow rate across the MV during the filling phase becomes higher with more ejected volume through the aortic valve. We also have a longer ejection duration, shorter iso-volumetric contraction time, and higher ejection fraction as a result of increasing *P*_endo_. On the other hand, if we do not apply *P*_endo,_ a much greater and non-physiological EDP is needed for the required ejection fraction. For example, with EDP=8 mmHg, the ejection fraction is only 29%. Only when EDP=20 mmHg, the pump function is comparable to the case with EDP=8 mmHg and *P*_endo_ = 16 mmHg. These results are summarised in Table 2.

## 4. Discussion

This study demonstrates the feasibility of integrating a MV model with a LV model from a healthy volunteer based on in vivo CMR images. This is the first physiologically based MV-LV model with fluid-structure interaction that includes nonlinear hyperelastic constitutive modelling of the soft tissue. The coupled MV-LV model is used to simulate MV dynamics, LV wall deformation, myocardial active contraction, as well as intraventricular flow. The modelling results are in reasonable quantitative agreement with in vivo measurements and clinical observations, such as the peak aortic flow rate (468mL/s vs. 498mL/s), the ejection duration (243 ms vs. 300 ms), peak cuff-measured systolic pressure (162 mmHg vs. 150 mmHg), and LV ejection fraction (51% vs. 57%). Note that the above comparisons are made with computational vs. imaged values.

Diastolic heart failure is usually associated with impaired myocardial relaxation and increased filling pressure [47,48]. In this study, we model the effects of myocardial relaxation by applying an endocardial surface pressure *P*_endo._ Specifically, we can enhance or suppress the myocardial relaxation by adjusting *P*_endo_. Our results in Table 2 show that, with an enhanced myocardial relaxation, say, when *P*_endo_ ≥ 12 mmHg, there is more filling during diastole, compared to the cases when *P*_endo_ < 12 mmHg under the same EDP. This in turn gives rise to higher ejection fraction and stroke volume. However, if myocardial relaxation is suppressed, diastolic filling is less efficient, with subsequently smaller ejection fraction and stroke volume. In the extreme case, when the myocardial relaxation is entirely absent, chamber volume increases by only 29.5 mL, and ejection fraction decreases to 29%. To maintain stroke volume obtained for *P*_endo_=12 mmHg, EDP needs to be as high as 20 mmHg. Indeed, increased EDP resulted from an impaired myocardial relaxation has been reported in a clinical study by Zile et al. [48]. A higher EDP indicates the elevated filling pressure throughout the refilling phase. Increased filling pressure can help to maintain a normal filling volume and ejection fraction, but runs the risks of ventricular dysfunction in the longer term, because pump failure will occur if no other compensation mechanism exists.

During diastole, the MV-LV model seems to yield a smaller orifice compared to the corresponding CMR images. In our previous study [25], the MV was mounted in a rigid straight tube, the peak diastolic filling pressure is around 10 mmHg, and the peak flow rate across the MV is comparable to the measured value (600 mL/s). While in this coupled MV-LV model, even though with additional *P*_endo,_ the peak flow rate (200mL/s) is much less than the measured value. One reason is because of the extra resistance from the LV wall, which is absent in the MV-tube model [25]. The diastolic phase can be divided into three phases [43]: the rapid filling, slow filling, and atrial contraction. During rapid filling, the transvalvular flow is resulted from myocardial relaxation (the “sucking” effect), which contributes to 80% of the total transvalvular flow volume. During slow filling and atrial contraction, the left atrium needs to generate a higher pressure to provide additional filling. In the coupled MV-LV model, the ramped pressure in the top plane of the inflow tract during late-diastole is related to the atrial contraction, and during this time, only 10% of the total transvalvular flow occurs. However, the peak flow rate in rapid filling phase is much lower compared to the measured value, which suggests that the myocardial relaxation would be much stronger.

In a series of studies based on in vitro *μ*CT experiments, Toma et al. [22,26,27] suggested that MV models with simplified chordal structure would not compare well with experimental data, and that a subject-specific 3D chordal structure is necessary. This may explain some of the discrepancies we observe here. A simplified chordal structure is used in this study because we are unable to reconstruct the chordal structure from the CMR data. CT imaging may allow the chordae reconstruction but it comes with radiation risk. Patient-specific chordal structure in the coupled MV-LV model would require further improvements of in vivo imaging techniques.

Several other limitations in the model may also contribute to the discrepancies. These include the uncertainty of patient-specific parameter identification, the uncertainty in MV geometry reconstruction from CMR images, the passive response assumption around the annulus ring and the valvular region of the LV model, and the lack of pre-strain effects. Studies addressing these issues are already under way. We expect that further improvement in personalised modelling and more efficient high performance computing would make the modelling more physiologically detailed yet fast enough for applications in risk stratification and optimisation of therapies in heart diseases.

## 5. Conclusion

Interaction between the mitral valve and the ventricular wall plays an essential role in cardiac pump function. In this study, we have developed a first fully coupled MV-LV model that includes fluid-structure interaction as well as soft tissue mechanics with model parameters determined from in vivo image data. The model geometry is derived from in vivo magnetic resonance images of a healthy volunteer, and incorporates three-dimensional finite element representations of the MV leaflets, sub-valvular apparatus, and the LV geometry. Fibre-reinforced hyperelastic constitutive laws are used to describe the passive response of the soft tissues, and a myofilament model is used to model the myocardial active contraction. Our results show that the developed MV-LV model can simulate MV-LV interaction with good agreements, including the peak aortic flow rate, the peak systemic pressure, the systolic ejection duration and the LV ejection fraction, with in vivo measurements despite several modelling limitations. We further find that with impaired myocardial active relaxation, the diastolic filling pressure needs to increase significantly in order to maintain a normal cardiac output, which is consistent with clinical observations in patients with impaired myocardial relaxation. This model thereby represents a step towards a whole-heart multiphysics modelling with a target for clinical applications.

## Figures and Tables

**Table 1:** Material parameter values for MV leaflets, chordae and the myocardium.

MV leaflets	*C*_1_ (kPa)	*a*_v_ (kPa)	*b*_v_ (kPa)
Anterior	17.4	31.3	55.93
Posterior	10.2	50	63.48
Chordae	*C* (kPa)		
Systole	9000		
Diastole	540		

Myocardium								
Passive	*a* (kPa)	*b*	*a*_f_ (kPa)	*b*_f_	*a*_s_ (kPa)	*b*_s_	*a*_8fs_ (kPa)	*b*_8fs_
	0.24	5.08	1.46	4.15	0.87	1.6	0.3	1.3
Active	*T*^req^ = 225 kPa							

**Table 2:** Effects of EDP and the endocardial pressure loading (*P*_endo_) on MV and LV dynamics. The case for sections 3.1–3.3 is highlighted with bold fonts.

Cases (mmHg)	*t*^iso–con^ (ms)	*t*^ejection^ (ms)	$V_{\text{LV}}^{\text{ejection}}\left(\text{mL}\right)$	$V_{\text{MV}}^{\text{filling}}\left(\text{mL}\right)$	$F_{\text{MV}}^{\text{peak}}\left(\text{mL}/\text{s}\right)$	LVEF (%)
EDP=8, *P*_endo_=8	60	227	52	60.6	412.93	47
EDP=8, *P*_endo_=10	58	237	57.6	65.9	442.60	49
**EDP=8**, *P*_endo_=**12**	57	243	63.2	72.1	468.41	51
EDP=8, *P*_endo_=14	55	251	67.8	76.8	486.84	53
EDP=8, *P*_endo_=16	54	256	72.3	81.3	503.76	54
EDP=8, *P*_endo_=0	75	174	20.8	29.5	209.54	29
EDP=12,*P*_endo_=0	64	213	41.0	50.9	343.47	42
EDP=14,*P*_endo_=0	61	226	50.6	59.8	406.81	47
EDP=16,*P*_endo_=0	58	243	61.8	71.9	459.64	51
EDP=18,*P*_endo_=0	56	251	68.5	79.3	486.58	54
EDP=20,*P*_endo_=0	55	262	75.7	86.2	511.16	55

## References

[1] Go AS, Mozaffarian D, Roger VL, Benjamin EJ, Berry JD, Blaha MJ, Heart disease and stroke statistics-2014 update. Circulation 2014;129(3):e28–e292. 24352519 10.1161/01.cir.0000441139.02102.80 PMC5408159

[2] Sacks MS, David Merryman W, Schmidt DE. On the biomechanics of heart valve function. J Biomech 2009;42(12):1804–24. 19540499 10.1016/j.jbiomech.2009.05.015 PMC2746960

[3] Votta E, Le TB, Stevanella M, Fusini L, Caiani EG, Redaelli A, Toward patient-specific simulations of cardiac valves: state-of-the-art and future directions. J Biomech 2013;46(2):217–28. 23174421 10.1016/j.jbiomech.2012.10.026 PMC3552085

[4] Sun W, Martin C, Pham T. Computational modeling of cardiac valve function and intervention. Annu Rev Biomed Eng 2014;16:53–76. 24819475 10.1146/annurev-bioeng-071813-104517 PMC5481457

[5] Kheradvar A, Groves EM, Dasi LP, Alavi SH, Tranquillo R, Grande-Allen KJ, Emerging trends in heart valve engineering: part I. Solutions for future. Ann Biomed Eng 2015;43(4):833–43. 25488074 10.1007/s10439-014-1209-z

[6] Kunzelman KS, Cochran R. Stress/strain characteristics of porcine mitral valve tissue: parallel versus perpendicular collagen orientation. J Cardiac Surg 1992:7(1):71–8. 10.1111/j.1540-8191.1992.tb00777.x 1554980

[7] Dahl SK, Vierendeels J, Degroote J, Annerel S, Hellevik LR, Skallerud B. FSI simulation of asymmetric mitral valve dynamics during diastolic filling. Comput Methods Biomech Biomed Eng 2012;15(2):121–30. 10.1080/10255842.2010.517200 21086206

[8] Weinberg EJ, Shahmirzadi D, Kaazempur Mofrad MR. On the multiscale modeling of heart valve biomechanics in health and disease. Biomech Model Mechanobiol 2010;9(4):373–87. 20066464 10.1007/s10237-009-0181-2

[9] Wang Q Sun W Finite element modeling of mitral valve dynamic deformation using patient-specific multi-slices computed tomography scans. Ann Biomed Eng 2013;41(1):142–53. 22805982 10.1007/s10439-012-0620-6

[10] Prot V, Skallerud B. Nonlinear solid finite element analysis of mitral valves with heterogeneous leaflet layers. Comput Mech 2009;43(3):353–68.

[11] Stevanella M, Maffessanti F, Conti CA, Votta E, Arnoldi A, Lombardi M, Mitral valve patient-specific finite element modeling from cardiac MRI: application to an annuloplasty procedure. Cardiovasc Eng Technol 2011:2(2):66–76.

[12] Lee C-H, Carruthers CA, Ayoub S, Gorman RC, Gorman JH, Sacks MS. Quantification and simulation of layer-specific mitral valve interstitial cells deformation under physiological loading. J Theor Biol 2015:373:26–39. 25791285 10.1016/j.jtbi.2015.03.004 PMC4404233

[13] Einstein DR, Del Pin F, Jiao X, Kuprat AP, Carson JP, Kunzelman KS, Fluid-structure interactions of the mitral valve and left heart: comprehensive strategies, past, present and future. Int J Numer Methods Biomed Eng 2010; 26(3–4):348–80. 10.1002/cnm.1280 PMC2864615 20454531

[14] Gao H, Qi N, Feng L, Ma X, Danton M, Berry C, Modelling mitral valvular dynamics–current trend and future directions. Int J Numer Methods Biomed Eng 2016. doi:10.1002/cnm.2858. PMC5697636 27935265

[15] Wenk JF, Zhang Z, Cheng G, Malhotra D, Acevedo-Bolton G, Burger M, First finite element model of the left ventricle with mitral valve: insights into ischemic mitral regurgitation. Ann Thoracic Surg 2010;89(5):1546–53. 10.1016/j.athoracsur.2010.02.036 PMC2887313 20417775

[16] Wong VM, Wenk JF, Zhang Z, Cheng G, Acevedo-Bolton G, Burger M, The effect of mitral annuloplasty shape in ischemic mitral regurgitation: a finite element simulation. Ann Thoracic Surg 2012;93(3):776–82. 10.1016/j.athoracsur.2011.08.080 PMC3432639 22245588

[17] Baillargeon B, Costa I, Leach JR, Lee LC, Genet M, Toutain A, Human cardiac function simulator for the optimal design of a novel annuloplasty ring with a sub-valvular element for correction of ischemic mitral regurgitation. Cardiovasc Eng Technol 2015:6(2):105–16. 25984248 10.1007/s13239-015-0216-z PMC4427655

[18] Einstein DR, Reinhall P, Nicosia M, Cochran RP, Kunzelman K. Dynamic finite element implementation of nonlinear, anisotropic hyperelastic biological membranes. Comput Methods Biomech Biomed Eng 2003:6(1):33–44. 10.1080/1025584021000048983 12623436

[19] Einstein DR, Kunzelman KS, Reinhall PG, Nicosia MA, Cochran RP. Non-linear fluid-coupled computational model of the mitral valve. J Heart Valve Dis 2005;14(3):376–85. 15974533

[20] Kunzelman K, Einstein DR, Cochran R. Fluid-structure interaction models of the mitral valve: function in normal and pathological states. Philos Trans R Soc B: Biol Sci 2007:362(1484):1393–406. 10.1098/rstb.2007.2123 PMC2440403 17581809

[21] Lau K, Diaz V, Scambler P, Burriesci G. Mitral valve dynamics in structural and fluid-structure interaction models. Med Eng Phys 2010;32(9):1057–64. 20702128 10.1016/j.medengphy.2010.07.008 PMC2989441

[22] Toma M, Einstein DR, Bloodworth CH, Cochran RP, Yoganathan AP, Kunzelman KS. Fluid-structure interaction and structural analyses using a comprehensive mitral valve model with 3D chordal structure. Int J Numer Methods Biomed Eng 2016. doi:10.1002/cnm.2815. PMC5183567 27342229

[23] Watton PN, Luo XY, Yin M, Bernacca GM, Wheatley DJ. Effect of ventricle motion on the dynamic behaviour of chorded mitral valves. J Fluids Struct 2008:24(1):58–74.

[24] Ma X, Gao H, Griffith BE, Berry C, Luo X. Image-based fluid-structure interaction model of the human mitral valve. Comput Fluids 2013;71:417–25.

[25] Gao H, Ma X, Qi N, Berry C, Griffith BE, Luo XY. A finite strain nonlinear human mitral valve model with fluid-structure interaction. Int J Numer Methods Biomed Eng 2014;30(12):1597–613. 10.1002/cnm.2691 PMC4278556 25319496

[26] Toma M, Jensen MØ, Einstein DR, Yoganathan AP, Cochran RP, Kunzelman KS. Fluid-structure interaction analysis of papillary muscle forces using a comprehensive mitral valve model with 3D chordal structure. Ann Biomed Eng 2016;44(4):942–53. 26183963 10.1007/s10439-015-1385-5 PMC4715994

[27] Toma M, Bloodworth CH, Pierce EL, Einstein DR, Cochran RP, Yoganathan AP, Fluid-structure interaction analysis of ruptured mitral chordae tendineae. Ann Biomed Eng 2016:1–13. doi:10.1007/s10439-016-1727-y. 27624659 PMC5332285

[28] Nash MP, Hunter PJ. Computational mechanics of the heart. J Elasticity Phys Sci Solids 2000:61(1–3):113–41.

[29] Chen WW, Gao H, Luo XY, Hill NA. Study of cardiovascular function using a coupled left ventricle and systemic circulation model. J Biomech 2016:49(12):2445–54. doi:10.1016/j.jbiomech.2016.03.009. 27040388 PMC5038162

[30] Quarteroni A, Lassila T, Rossi S, Ruiz-Baier R. Integrated heart-coupling multiscale and multiphysics models for the simulation of the cardiac function. Comput Methods Appl Mech Eng 2016;314:345–407.

[31] Rausch MK, Zöllner AM, Genet M, Baillargeon B, Bothe W, Kuhl E. A virtual sizing tool for mitral valve annuloplasty. Int J Numer Methods Biomed Eng 2016. doi:10.1002/cnm.2788. PMC5289896 27028496

[32] Peskin CS. Flow patterns around heart valves: a numerical method. J Comput Phys 1972;10(2):252–71.

[33] McQueen DM, Peskin CS, Yellin EL. Fluid dynamics of the mitral valve: physiological aspects of a mathematical model. Am J Physiol Heart Circ Physiol 1982;242(6):H1095–110. 10.1152/ajpheart.1982.242.6.H1095 7091349

[34] Peskin CS. Numerical analysis of blood flow in the heart J Comput Phys 1977;25(3):220–52.

[35] Peskin CS. The immersed boundary method. Acta Numer 2002:11:479–517.

[36] Yin M, Luo XY, Wang TJ, Watton PN. Effects of flow vortex on a chorded mitral valve in the left ventricle. Int J Numer Methods Biomed Eng 2010;26(3–4):381–404.

[37] Chandran KB, Kim H. Computational mitral valve evaluation and potential clinical applications. Ann Biomed Eng 2015;43(6):1348–62. 25134487 10.1007/s10439-014-1094-5 PMC4334752

[38] Gao H, Wang H, Berry C, Luo XY, Griffith BE. Quasi-static image-based immersed boundary-finite element model of left ventricle under diastolic loading. Int J Numer Methods Biomed Eng 2014. doi:10.1002/cnm.2652. PMC4233956 24799090

[39] Griffith BE, Luo XY Hybrid finite difference/finite element version of the immersed boundary method. International Journal for Numerical Methods in Biomedical Engineering, in press, DOI:10.1002/cnm.2888. PMC5650596 28425587

[40] Gao H, Berry C, Luo XY. Image-derived human left ventricular modelling with fluid-structure interaction In: Functional imaging and modeling of the heart Springer; 2015 p. 321–9.

[41] Holzapfel GA, Ogden RW. Constitutive modelling of passive myocardium: a structurally based framework for material characterization. Philos Trans R Soc Lond A: Math Phys Eng Sci 2009;367(1902):3445–75. 10.1098/rsta.2009.0091 19657007

[42] Niederer S, Hunter P, Smith N. A quantitative analysis of cardiac myocyte relaxation: a simulation study. Biophys J 2006;90(5):1697–722. 16339881 10.1529/biophysj.105.069534 PMC1367320

[43] Nishimura RA, Tajik AJ. Evaluation of diastolic filling of left ventricle in health and disease: Doppler echocardiography is the clinician’s Rosetta stone. J Am Coll Cardiol 1997:30(1):8–18. 9207615 10.1016/s0735-1097(97)00144-7

[44] Laniado S, Yellin E, Kotler M, Levy L, Stadler J, Terdiman R. A study of the dynamic relations between the mitral valve echogram and phasic mitral flow. Circulation 1975:51 (1):104–13. 1109311 10.1161/01.cir.51.1.104

[45] Griffith BE. Immersed boundary model of aortic heart valve dynamics with physiological driving and loading conditions. Int J Numer Methods Biomed Eng 2012:28(3):31–45. 10.1002/cnm.1445 25830200

[46] Mangion K, Gao H, McComb C, Carrick D, Clerfond G, Zhong X, A novel method for estimating myocardial strain: assessment of deformation tracking against reference magnetic resonance methods in healthy volunteers. Sci Rep 2016;6:38774. doi:10.1038/srep38774. 27941903 PMC5150576

[47] Hay I, Rich J, Ferber P, Burkhoff D, Maurer MS. Role of impaired myocardial relaxation in the production of elevated left ventricular filling pressure. Am J Physiol Heart Circ Physiol 2005;288(3):H1203–8. 15498827 10.1152/ajpheart.00681.2004

[48] Zile MR, Baicu CF, Gaasch WH. Diastolic heart failure-abnormalities in active relaxation and passive stiffness of the left ventricle. New Engl J Med 2004; 350(19):1953–9. 15128895 10.1056/NEJMoa032566

